# Alternation of Neuronal Feature Selectivity Induced by Paired Optogenetic-Mechanical Stimulation in the Barrel Cortex

**DOI:** 10.3389/fncir.2021.708459

**Published:** 2021-09-01

**Authors:** Yu-Po Cheng, Jian-Jia Huang, Chun-I Yeh, Yu-Cheng Pei

**Affiliations:** ^1^Department of Physical Medicine and Rehabilitation, Linkou Chang Gung Memorial Hospital, Taoyuan, Taiwan; ^2^Department of Psychology, College of Science, National Taiwan University, Taipei, Taiwan; ^3^Center of Vascularized Tissue Allograft, Linkou Chang Gung Memorial Hospital, Taoyuan, Taiwan; ^4^School of Medicine, College of Medicine, Chang Gung University, Taoyuan, Taiwan; ^5^Healthy Aging Research Center, Chang Gung University, Taoyuan, Taiwan

**Keywords:** barrel cortex, single unit, neuroplasticity, optogenetic, neuromodulation

## Abstract

Paired stimulation has been applied to modulate neuronal functions in the primary somatosensory cortex but its utility in the alternation of tuning function, such as direction tuning for whisker stimuli, remains unclear. In the present study, we attempted to manipulate feature preferences in barrel cortical neurons using repetitive paired whisker deflection combined with optogenetic stimulation and to obtain optimal parameters that can induce neuroplasticity. We found no significant response changes across stimulus parameters, such as onset asynchronies and paired directions. Only when paired stimulation was applied in the nonpreferred direction of the principal whisker of a neuron, were the neuron’s responses enhanced in that direction. Importantly, this effect was only observed when the optogenetic stimulus preceded the mechanical stimulus. Our findings indicate that repetitive paired optogenetic-mechanical stimulation can induce *in vivo* neuroplasticity of feature selectivity in limited situations.

## Introduction

The induced neuroplasticity of sensory function using repetitive paired stimulation has been observed in the brain. Previous studies revealed that a neuron’s response strength and feature selectivity could be altered by paired peripheral and cortical stimulations, with most of the evidence coming from the visual (Yao and Dan, 2001; Meliza and Dan, 2006; Li and DiCarlo, 2008; El-Boustani et al., 2018) and auditory (Kilgard and Merzenich, 1998; Kilgard et al., 2001; Froemke et al., 2007; Shetake et al., 2012; Borland et al., 2019) systems. Although adaptation induced by repetitive whisker stimulation in the whisker-barrel circuits has been shown to induce alternation of stimulus-driven activities in single cell (Ganmor et al., 2010; Mohar et al., 2013, 2015; Katz and Lampl, 2021) and neural population (Adibi et al., 2013), it remains unclear whether the feature selectivity can be altered by repetitive paired stimulation. It is thus important to characterize the feasibility of inducing functional plasticity in somatosensation *via* repetitive paired stimulation.

Rodent’s primary somatosensory cortex barrel field (S1BF) is a feasible model for studying stimulus-induced neuroplasticity because of its familiar anatomical and functional organization (Woolsey and Van der Loos, 1970; Van der Loos and Woolsey, 1973; Armstrong-James et al., 1992; Petersen, 2007; Feldmeyer, 2012; Feldmeyer et al., 2013; Adibi, 2019; Petersen, 2019; Staiger and Petersen, 2021), properties of angular tuning (Bruno et al., 2003; Andermann and Moore, 2006; Li and Ebner, 2007; Tsytsarev et al., 2010; Kwon et al., 2018), and experimental convenience. The whisker-barrel system is characterized by its one-to-one topographic relationship and is thus widely used in the evaluation of functional plasticity (Katz et al., 2006; Heiss et al., 2008; Jacob et al., 2012, 2017) and whisker-map reorganization (Van der Loos and Woolsey, 1973; Fox, 2002; Petersen et al., 2003; Feldman and Brecht, 2005; Feldmeyer et al., 2013; Adibi, 2019; Petersen, 2019).

Despite the popularity of this model, only a handful of studies have examined alternations in neuronal function using paired peripheral and cortical stimulations of the S1BF. Two studies revealed the possibility of response strength alterations in the somatosensory system that seemed relevant to the sequence of stimulus modality in their pairs. Jacob et al. (2007) found that paired whisker stimulation and current injection into S1BF could suppress stimulus-driven neural activities in S1BF when the latter was delivered before the former. In a later study, Gambino and Holtmaat (2012) reported the facilitation of stimulus-driven post-synaptic potential in S1BF after applying paired principal whisker (PW) stimulation before current injection. However, much of the interpretation was based on membrane potential results, hence their influence on the spike output of multiple neurons is uncertain.

Based on the fact that paired stimulation of excitatory neuron and peripheral sensory inputs could alter feature preference (Meliza and Dan, 2006; Jacob et al., 2007), we designed an experiment using cell-type-specific optogenetic stimulation and whisker deflection pairs. Optogenetics provides cell-type-specific control, a property that has been utilized to decrease angular tuning in the barrel cortex (Pauzin et al., 2019). In the present study, we attempted to test the feasibility of using paired optogenetic and mechanical stimulation to modify the feature selectivity of S1BF neurons *in vivo* and ascertain the optimal parameters to achieve this goal. To characterize the neuroplasticity of feature selectivity in a neuronal assembly, we presented paired optogenetic and mechanical stimulation delivered through custom-designed piezoelectric-actuator-based stimulators. We hypothesized that a neuron’s feature selectivity can be altered when the paired stimulation is applied to a neuron’s preferred features, such as its preferred whisker direction.

## Materials and Methods

### Animals

This study included 17 male adult Sprague-Dawley rats aged 7–8 weeks and weighing 250–300 g, which were obtained from BioLASCO Taiwan (Taipei, Taiwan). The animals were housed in a 12-h light/12-h dark circadian cycle at room temperature of 22–25°C, with food and water available *ad libitum*. All animal procedures were performed in accordance with the regulations of the Institutional Animal Care and Use Committee of Linkou Chang Gung Memorial Hospital.

### Stereotaxic Injection of Virus Vector

The animal was first sedated with a mixture of air and isoflurane (3%). Anesthesia was then inducted with an intraperitoneal injection of a mixture of ketamine (100 mg/kg) and xylazine (10 mg/kg). Anesthesia depth was maintained by one-third of the induction dosage delivered every 30 min and monitored so that no pain-elicited withdrawal reflex could be observed during the surgery.

The animal’s hair overlying the head was removed, and the animal was placed on the stereotaxic frame. Body temperature was maintained at 37°C using a heating pad. Topical analgesia (2% lidocaine ointment) was applied to the scalp before the first incision. An incision was made in the skin above the skull, and then tissues on the skull were removed. For virus injection, a burr hole was created in the skull overlying the right S1BF (AP: −2 mm, ML: +5 mm) using a drill. The rAAV9-CaMKII-hChR2(E123A)-mCherry-WPRE-hGH virus from Penn Vector Core (Philadelphia, PA, USA) was loaded into a microinjection syringe (701RN, Hamilton, NV, USA), which was slowly inserted to the target area 1.5 mm below the brain surface. Ten minutes after reaching the injection site, 0.7 μl of the virus was injected at an injection rate of 0.07 μl/min. Ten minutes after the injection, the syringe was withdrawn slowly, the skin was closed with sutures, and finally, topical analgesia (2% lidocaine ointment) was applied.

### Electrophysiological Recording and Optogenetic Stimulation

The formal experiment was conducted 4 weeks after the virus injection. The animal was first sedated with a mixture of air and isoflurane (3%). Anesthesia was then inducted using urethane (1.4 g/kg, i.p.) and maintained using one-third of the induction dosage every 3–4 h (Casas-Torremocha et al., 2017). After removal of the scalp, three fixation screws were placed, with one at the left parietal bone (AP: −4 mm, ML: −4 mm) and two at bilateral occipital bones (AP: −16 mm, ML: −2.5, and +2.5 mm), to which a copper pillar was connected to fixate the head. The grounding cables were wired on the three fixation screws, and dental cement was applied to secure the exposed skull, screws, pillar, and grounding cables.

**Figure 1:**
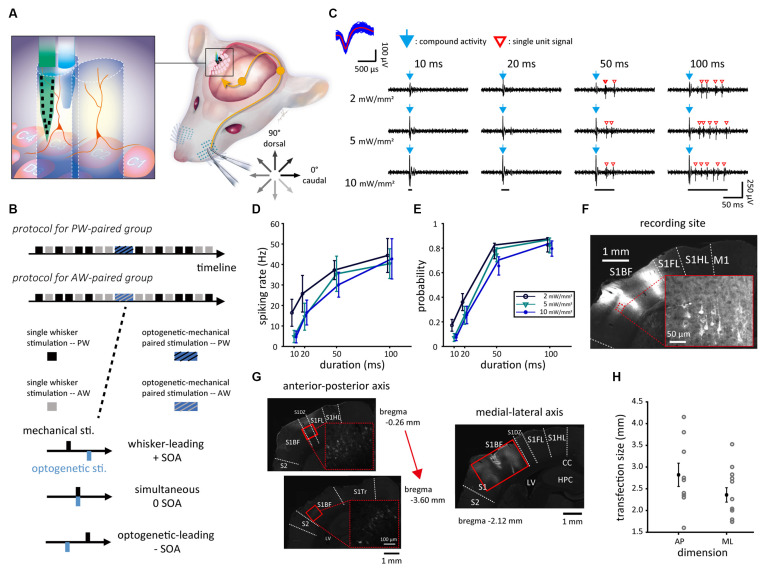
Experimental setup and neuronal responses to mechanical, optogenetic, and paired stimulations. **(A)** The experimental setup of single-unit recording in S1BF *via* the probe-fiber bundle when presenting mechanical (whisker), optogenetic, or paired stimulations. During mechanical stimulation, the whisker was deflected in one of the eight directions. The tactile afferent signals were relayed to the ipsilateral brainstem and ascended to the contralateral thalamus then the S1. The left panel shows the experimental setup in which CaMKII-mCherry-expressing neurons (red) were optogenetically stimulated by laser light delivered into S1BF *via* an optical fiber (blue) that bundled with the electrode probe (green). **(B)** The experiment consists of the prepaired recording, paired stimulation, and postpaired recording. The SOAs were −40 to 40 ms in 10-ms steps. **(C)** In a factorial design with four stimulation durations by three stimulation intensities, the duration was 10, 20, 50, or 100 ms and the intensity was 2, 5, or 10 mW/mm^2^. The responses of sample single unit (top left) isolated from the recorded spike trains during optogenetic stimulations showed compound activities (blue arrows) as a prompt response to the onset of optogenetic stimulation (bottom) and a series of isolated single-unit responses (red empty arrowheads). **(D)** Data averaged from 17 single units obtained from the four animals showed that the spiking rate monotonously increased with the stimulus duration and was relatively weakly affected by the stimulus intensity. **(E)** A similar trend was also found in firing probability. **(F)** The fluorescence image showed that rAAV-transfected neurons, which expressed mCherry (in the magnification view with red outlines), located in S1BF. The disconnected area of mCherry fluorescence in S1BF was the recording site, which showed the tissue change induced by the insertion of the probe-fiber bundle. **(G)** Neurons transfected with rAAV expressed mCherry (highlighted in high magnification views marked in dashed red squares), locating mainly in S1BF as shown in the coronal brain slices in an anterior-to-posterior order. Solid red rectangles represent the transfection area, in which mCherry-expressing cell bodies can be observed. **(H)** The size of the transfection area measured in the anterior-posterior (AP) and medial-lateral (ML) dimensions. Error bars represent the mean ± s.e.m. and dots represent data for animals. S1, primary somatosensory cortex; S1BF, S1 barrel field; S1DZ, S1 dysgranular zone; S1FL, S1 forelimb region; S1HL, S1 hindlimb region; S1Tr, S1 trunk region; S2, secondary somatosensory cortex; CC, corpus callosum; LV, lateral ventricle; HPC, hippocampus; D3V, dorsal third ventricle; SOA, stimulus onset asynchrony.

Craniotomy with a 4 × 4 mm^2^ window (centered at AP: −3 mm, ML: +5 mm) was performed for the single-unit recording in S1BF. The 16-channel silicon-based electrode probe (E16 electrodes, Cambridge NeuroTech, Cambridge, UK) was used for recording. We parallelly bundled a tapered-tip optical fiber (outside diameter: 200 μm; material: ferrule fiber stubs; Hong Kong Plexon, Beijing, PRC) with the silicon probe at a fiber-to-probe orthogonal distance of 400 μm for performing optogenetic stimulation during single-unit recording (Figure 1A). After removing the dura mater, the probe-fiber bundle was slowly inserted *via* a direction perpendicular to the brain surface of S1BF and reached a depth of 1,200 μm.

Optogenetic stimulation was mediated by a blue-light laser with a wavelength of 473 nm, intensity of 5–10 mW/mm^2^, and duration of 50 ms. The light path started from a laser source (PSU-H-LED, MBL-F-473-200 mW, Changchun New Industries Optoelectronics Technology, Changchun, PRC) delivered through an externally controlled shutter (LS2S2T0, Vincent Associates, NY, USA), and it ended up projecting into S1BF *via* the optical fiber. Neural spike trains were recorded by the Blackrock multichannel recording system (Cerebus, Blackrock Microsystems, UT, USA) with a sampling rate of 30 kHz and bandpass filter from 250 Hz to 7.5 kHz.

To probe the optimal parameter for optogenetic stimulation, a factorial-designed testing, as a pilot study, of 17 units from S1BF in four animals was conducted. Blue light stimulation was applied with one of three optical intensities (2, 5, or 10 mW/mm^2^) and one of four optical stimulation durations (10, 20, 50, or 100 ms), yielding a total of 12 (3 × 4) parameter combinations delivered in a pseudorandom order (Figure 1B). Each parameter combination was applied in a block with 20 consecutive repetitions in 1 Hz. Blocks were interleaved with 3-min breaks that allowed for the recovery of neuronal function to its baseline. Spontaneous neuronal activities were recorded for 20 s before and after this experiment.

### Paired Optogenetic-Mechanical Stimulation

All whiskers were trimmed to 10 mm in length to fit into the whisker stimulation apparatus before the experiment. Single units were assigned to receive paired stimulation in either the PW-paired or AW-paired group (Figure 1B, top). Neuronal responses evoked by both PW and adjacent whisker (AW) stimulations, in eight directions, were measured separately. The parameters of paired stimulation experiments included the paired whisker, stimulus onset asynchrony (SOA), and paired direction.

For every single unit, PW was defined as the whisker that evoked the highest spiking rate in manual mapping, and later confirmed by online peri-stimulus time histogram (PSTH) analysis; AW was one of the surrounding whiskers (the eight nearby whiskers in a three by three grid centered with the PW; e.g., PW = C2, then nearby whiskers = B1–3, C1, C3, and D1–3) that showed the highest elicited spiking rate. Each of the PW and AW was inserted into a piezoelectric-actuator-based whisker stimulator (Pei et al., 2019; Figure 1A). From data delineated in Figures 1D,E, optogenetic stimulation parameters were set as intensity = 5–10 mW/mm^2^ and duration = 50 ms, for most units could be elicited with precisely timed and effective responses.

**Figure 2:**
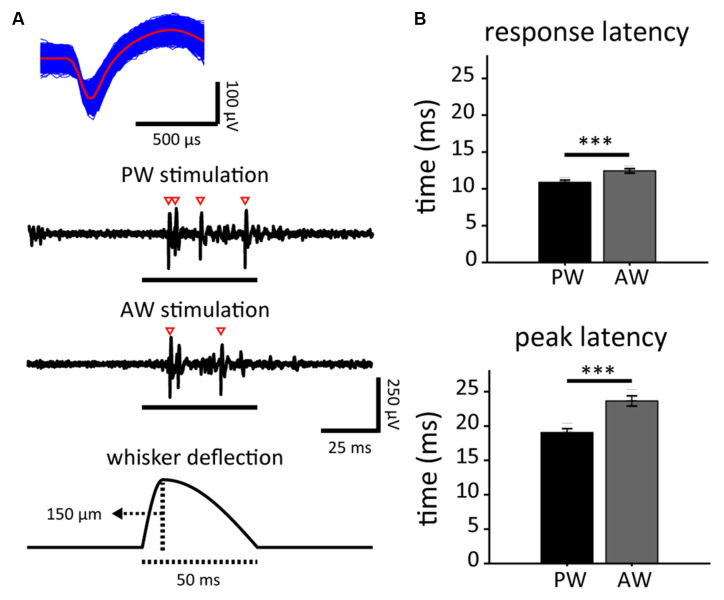
Stimulus-driven activities in response to principal whisker (PW) and adjacent whisker (AW) stimulations, and the spontaneous neuronal activities in neuron populations. **(A)** The spike waveform of an example single unit (top) isolated from the spike trains as a response to stimuli presented to PW and AW (middle). Spikes are marked by empty arrowheads. Each whisker stimulus consisted of a 9-ms deflection and 41-ms restoration (bottom). **(B)** PW stimulation-induced shorter response latencies (top: response latency; bottom: peak latency; ***p < 0.001, Wilcoxon signed-rank test).

The formal experiment consisted of electrophysiological recordings under the prepaired stimulation, one session of paired optogenetic-mechanical stimulation, and postpaired stimulation. Each of the electrophysiological recordings before and after the paired stimulations had eight sessions, four for the PW and AW stimulation, respectively (Figure 1B, top). For each trial, the whisker was deflected (9-ms rise time and 41-ms decay time; Figure 2A) in one of the eight directions, ranging from 0° (caudal in Figure 1A) to 315° in 45° steps. Each block consisted of eight trials corresponding to the eight directions and one blank trial (no stimulation) in a pseudorandom order. Each session had 20 blocks, yielding 180 trials. The inter-trial-interval and the inter-block-interval were both 75 ms, and the inter-session-interval was 20 s.

Paired optogenetic-mechanical stimulation was delivered with a variety of temporal sequences of the paired stimuli (Figure 1B, top). Specifically, the time interval between the optogenetic and mechanical stimulations was defined as the SOA, ranging from −40 to 40 ms in 10-ms steps, according to the following equation:

(1) SOA=optogenetic stimulation onset time - mechanical stimulation onset time

such that positive SOAs indicated mechanical stimulation led optogenetic stimulation, zero SOA simultaneous stimulation, and negative SOAs optogenetic stimulation led mechanical stimulation (Figure 1B, bottom). Every single unit could only receive one session of paired stimulation, which consisted of 100 trials (Jacob et al., 2007) and 950-ms inter-trial-interval respective to the 50-ms mechanical stimulation.

The relationship between the whisker deflecting direction used in the paired stimulation and the preferred direction of a single unit was characterized. The preferred direction was defined as the direction in which whisker stimulation evoked a single unit’s highest spiking rate among the eight deflecting directions. Accordingly, the three relative direction conditions were defined as: (1) the preferred direction condition, in which the paired direction was within ±45° of the neuron’s preferred direction; (2) the orthogonal direction condition, in which the paired direction was orthogonal to the neuron’s preferred direction; and (3) the nonpreferred direction condition, in which the paired direction was within ±45° of the direction opposite to the neuron’s preferred direction. For the SOA analysis, several SOAs were grouped as: (1) the optogenetic-leading condition (SOA = −40, −30, −20, or −10 ms); and (2) the whisker-leading condition (SOA = 10, 20, 30, or 40 ms). This analysis thus yielded a three (paired direction condition) by two (stimulus order condition) factorial design.

Two additional control groups were conducted in which repetitive stimulation of one modality was applied instead of repetitive paired stimulation of two modalities. The optogenetic-only group received the optogenetic protocol that was identical to that used in the paired stimulation except that no whisker stimulation was delivered. Analogously, the mechanical-only group received the mechanical protocol that was identical to that used in the paired stimulation except that no optogenetic stimulation was delivered. The whisker stimulation can be presented in one of the PW and AW.

In summary, single units were assigned to one of the five groups, including the paired stimulation (the PW-paired and AW-paired groups), mechanical-only (the PW-only and AW-only groups), and optogenetic-only group.

### Histology

After the experiment, the animals were euthanized with an overdose of sodium pentobarbital (100 mg/kg, i.p.) and then perfused with 200 ml of phosphate-buffered saline (PBS, 1×, pH 7.4) and 4% paraformaldehyde. The brain was removed from the skull, soaked in fixative for 24 h at 4°C, and transferred to a solution of 30% sucrose for 3 days. The brain was frozen and sectioned along the coronal plane at a thickness of 50 μm using a freezing microtome at −20°C.

Cells with the CaMKII promoter could be transfected by the viral vector and thus expressed ChR2 and mCherry fluorescent protein. The fluorescence image was obtained using a fluorescence microscope with a CCD camera (Leica Microsystems, Wetzlar, Germany). The anterior-posterior position of each slice was further confirmed by comparing landmark structures according to the atlas (Paxinos and Watson, 1998; Figures 1F,G). Additionally, the transfection range of the viral vector was estimated on the anterior-posterior and medial-lateral axes by identifying the observable boundary of mCherry-expressing cell bodies (Figures 1G,H).

### Data Analysis

Single units were isolated using the Offline Sorter (Plexon, TX, USA). The spike trains were first filtered by a Butterworth high-pass filter (4-pole, pass band >250 Hz). A threshold was applied for spike detection with 3.5-fold of the standard deviation or by manual sorting. Single units were first identified from their spike shape space projected on axes of principal components one, two, and three using the principal component analysis (PCA) and clusters vs. time analysis. Auto-correlation and Cross-correlation functions were used to examine the degree to which each single unit cluster fits the definition of a putative single unit. The spikes with inter-spike-interval (ISI) less than 1-ms (the refractory period of spikes) were removed. Finally, the trough-to-peak spike width of every single unit was computed, from which each could be assigned as a putative narrow spiking or broad spiking neuron (Mitchell et al., 2007; Guo et al., 2014), and the results of these two types of putative neurons could be compared.

**Figure 3:**
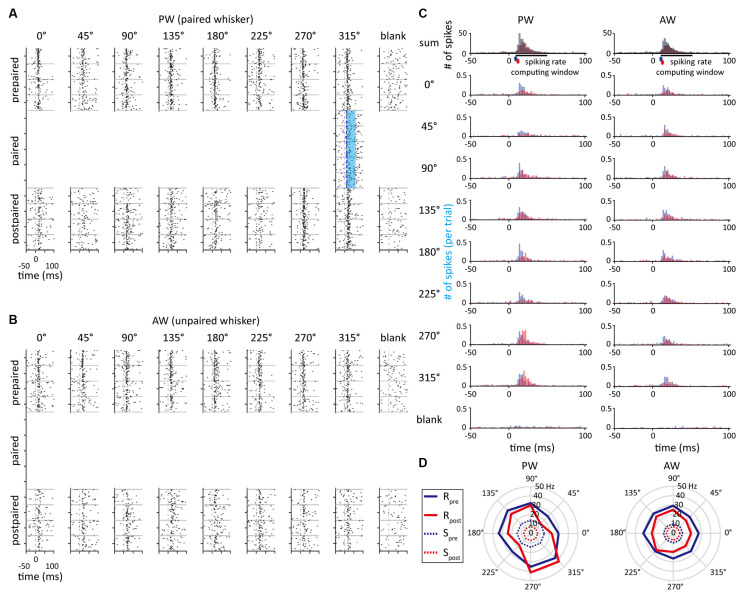
The example unit that showed an increased spiking rate in the paired direction. **(A)** Peri-event raster plots obtained from the single unit delineated in Figure 2A as a response to whisker stimulation in PW in the prepaired, paired, and postpaired periods. The blue area indicates the time period of optogenetic stimulation. **(B)** The unit’s peri-event raster plots as a response to whisker stimulation in AW. **(C)** Peri-stimulus time histograms (PSTHs) obtained from PW and AW stimulations. The asterisks represent the response onset. The blue and red histograms indicate neural responses before and after paired stimulations, respectively. **(D)** The polar plots represent the neural responses before (blue) and after (red) paired stimulations. Solid and dashed lines indicate spiking rates of stimulus-driven response (R) and spontaneous activity (S), respectively.

The data were analyzed using Matlab software (MathWorks, MA, USA). Raster plots (Figures 3A,B) and PSTHs (Figure 3C) were calculated within a 150-ms time window starting from 50 ms before stimulus onset (2-ms bin) to characterize the neuronal responses. The unresponsive units that showed no significant difference of mean spiking rate between the 50-ms time windows before and after stimulus onset (see Statistics) were omitted from the further analysis. Neuronal onset latency was defined as the period from the stimulus onset to the first bin of two continuous bins with activities higher than three-fold of the standard deviation (the SD was calculated in a 50-ms time window before stimulus onset) within a 100-ms time window after stimulus onset (Figure 3C). The peak latency was defined as the period from the stimulus onset to the highest bin within a 100 ms time window starting from the stimulus onset. If the peak latency was longer than 50 ms, the trial was considered an unresponsive trial.

The stimulus-driven spiking rate, ${\hat{R}}_{i}$, was computed by the mean spiking rate, *R_i_*, in a 50-ms time window following the onset latency, subtracted by the mean spiking rate of the blank trials, S, (Figures 3C,D) as

(2) $${\hat{R}}_{i}=R_{i}-S$$

where, *i* = 1–8, which denoted one of the eight whisker bending directions. The mean response across eight directions was also calculated to estimate the individual unit’s overall response to whisker stimulations.

In order to normalize the results for the neurons’ spiking rates, an approach that could avoid a biased result that was dominated by neurons with high spiking rates, the Change Index (*CI*) was developed to gauge the change of spiking rate in each direction after the paired stimulation:

(3) $$CI=\frac{{\hat{R}}_{post}-{\hat{R}}_{pre}}{{\hat{R}}_{post}+{\hat{R}}_{pre}}$$

where, ${\hat{R}}_{pre}$ and ${\hat{R}}_{post}$ represented the ${\hat{R}}_{i}$ in the prepaired and postpaired periods, respectively. The *CI* ranged from 1 to −1. The positive values of *CI* indicated the facilitation of neural response, and the negative values of *CI* indicated the suppression of neural response. For example, a *CI* value of 0.1 indicates a 10% increase of spiking rate based on its summation of prepaired and postpaired spiking rates. The *CI* of the mean response across eight directions was also calculated using Equation 3 with ${\hat{R}}_{i}$ replaced by the mean response.

### Statistics

Data were presented as the mean ± the standard error of the mean (s.e.m.). Nonparametric statistic methods were applied due to the scarcity (in some conditions) and inequality of data points among experiment conditions. The Wilcoxon signed-rank test was conducted to compare neuronal activities between the prepaired and postpaired measurements and the Wilcoxon rank-sum test was conducted to compare the *CI*s between paired direction conditions and between paired and control groups. The Spearman’s rank correlation was used for examining the relationship between the magnitude of feature selectivity, *DI* and *OI*, and the *CI*. Meanwhile, to verify that no biases were induced by arbitrarily assigning single units to the three paired direction conditions, the Kruskal-Wallis test was applied to compare single units’ original spontaneous spiking rate, evoked spiking rate, onset latency, and peak latency across the three paired direction conditions. The Kruskal-Wallis test was also conducted to test the effect of SOA and paired direction.

The Bonferroni correction was applied as we performed multiple comparisons. Accordingly, statistical significance was specifically defined as *p* < 0.05/9, 0.05/3, 0.05/2 in testing the effect of SOA, the effect of paired direction, and the effect of unpaired directions, respectively. The effect size of the Wilcoxon tests was estimated by the *r* value, which was calculated by dividing the *Z* value by the square root of the sample size (Rosenthal et al., 1994). The interpretation of *r* values was analogous to Cohen’s d, in which *r* values of 0.10, 0.30, and 0.50 indicate small, medium, and large effects, respectively (Cohen, 1992).

## Results

### Transfections of Viral Vector in the Neurons at Recording Sites

Both electrophysiological data of light-induced neuronal activity (Figures 1F,G) and histological inspection (Figures 1C,E) revealed successful transfections of viral vectors at recording sites.

A factorial design on light stimulation in four animals, as a pilot study, was conducted to probe the optimal parameter of light intensity and duration prior to the formal experiments. Although compound activities were robust and might mask initial responses (Figure 1F), Figures 1G,H showed that reliable light-induced neuronal responses occurred at 50-ms and 100-ms durations regardless of light intensity. Thus, to gain better temporal control of neuronal activities, we applied a 50-ms duration of light stimulation at a sufficient level of light intensity (5–10 mW) evoking action potentials for the recorded single units in the paired stimulation.

The recording sites were confirmed located in S1BF for all 17 animals. The transfection area estimated in the anterior-posterior (AP) and medial-lateral (ML) axes among 15 animals were 2,822 ± 268 and 2,458 ± 184 μm, respectively (Figure 1E). Based on the confirmation of successful optogenetic expression, we conducted repetitive stimulation pairs of mechanical and optogenetic stimuli attempting to induce neuroplasticity *in vivo*.

### Properties of Neuronal Activities in Response to Whisker Stimulation

Neuronal responses to whisker deflection were measured before and after the experiment in order to characterize any change caused by the paired stimulation. Extracellular recordings of S1BF single units were conducted in 17 male adult SD rats, resulting in 181 high-quality units responsive to whisker stimulations out of 462 recorded single units. Through online manual mapping and offline analysis, we defined the PW and AW of each recorded single unit based on stimulus-driven spiking rate. PW was defined as the whisker that evoked a recorded single unit’s greatest stimulus-driven response, while AW was defined as the surrounding whisker (of PW) that evoked the greatest response. Accordingly, the response onset latency of PW stimulation was shorter than that of AW stimulation (*p* < 0.001; Figure 2B, top). The response peak latency of PW was also shorter than that of AW (*p* < 0.001, Wilcoxon signed-rank test; Figure 2B, bottom). The results are compatible with the fact that PW response is the fastest among an S1BF neuron’s whisker receptive field (Armstrong-James et al., 1992). With the confirmation of reliable optogenetic and whisker responses from recorded single units, we paired the two modalities to induce *in vivo* neuroplasticity of S1BF neuronal tuning in response to PW and AW stimulations. Among the 181 high-quality units, 98 were in the PW-paired group and 83 in the AW-paired group.

### The Effect of Paired Stimulation

In order to measure the effect of paired optogenetic-mechanical stimulation on S1BF neuroplasticity and the influence of pairing conditions, the SOA, paired direction, and paired whisker were manipulated (also see “Materials And Methods”). Figure 3 shows a sample unit’s neuronal activities at the prepaired, paired, and postpaired periods, in which optogenetic stimulation was repetitively paired with mechanical stimulation to one whisker (the paired whisker) in one of the eight deflecting directions (the paired direction). This single unit was in a whisker-leading (SOA = 10 ms), preferred direction condition (the paired direction = the preferred direction = 315°) of the PW-paired group.

We first compared the stimulus-evoked spiking rates before and after pairing in all recorded single units. In the PW-paired group, PW (pre: 10.50 ± 1.07 Hz, post: 11.46 ± 1.24 Hz, *n* = 98, *p* = 0.648) and AW responses (pre: 13.50 ± 2.55 Hz, post: 12.30 ± 2.22 Hz, *p* = 0.058) did not alter after pairing (Table 1). In the AW-paired group, PW (pre: 10.00 ± 1.00 Hz, post: 7.50 ± 1.50 Hz, *n* = 83, *p* = 0.679) and AW responses (pre: 13.88 ± 2.38 Hz, post: 13.25 ± 0.92 Hz, *p* = 0.577) also did not alter after pairing. Analogously, *CI*s, the normalized spiking rate change index avoiding bias caused by individual extreme values (see “Materials And Methods” section), did not differ to zero (all *p* > 0.05) in all aforementioned conditions (Table 1), again indicating that paired stimulation did not alter stimulus-elicited spiking rates in these single units.

**Table 1:** Properties of neuronal responses before and after paired stimulation.

	PW-paired group				AW-paired group			
	PW (*n* = 98)		AW (*n* = 98)		PW (*n* = 83)		AW (*n* = 83)	
	Pre-	Post-	Pre-	Post-	Pre-	Post-	Pre-	Post-
Spiking rate (Hz)	10.50 ± 1.07	11.46 ± 1.24	13.50 ± 2.55	12.30 ± 2.22	10.00 ± 1.00	7.50 ± 1.50	13.88 ± 2.38	13.25 ± 0.92
*CI*	0.005 ± 0.033		−0.060 ± 0.047		0.005 ± 0.033		−0.060 ± 0.047	
Onset latency (ms)	11.05 ± 0.42	11.21 ± 0.43	11.94 ± 0.42	12.48 ± 0.40	10.68 ± 0.44	10.60 ± 0.44	13.04 ± 0.42	13.29 ± 0.43
Peak latency (ms)	11.83 ± 0.70	11.83 ± 0.68	12.05 ± 0.88	12.18 ± 1.03	9.96 ± 0.71	9.78 ± 0.63	12.12 ± 0.66	12.90 ± 0.71

We next compared the response latencies of before and after pairing. In the PW-paired group, the onset latencies and peak latencies for PW and AW stimulation did not alter after pairing (all *p* > 0.05; Table 1). In the AW-paired group, the onset latencies and peak latencies for PW and AW stimulation did not alter after pairing (all *p* > 0.05).

### The Influence of SOA on the Effect of Paired Stimulation

In order to separate the effect of each parameter, we analyzed the SOA, paired direction, and their combination in sequence. Furthermore, to reduce biased results due to individual differences of spiking rate among single units, we introduced *CI* (also see “Materials And Methods” section) to gauge the change of mechanical stimulus-driven responses between the prepaired and postpaired periods, which was a ratio of response change to response summation.

**Figure 4:**
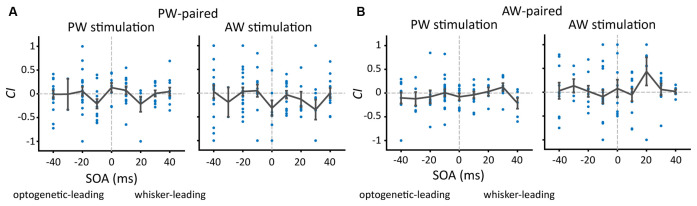
The proportion of units showing a change after paired stimulation and the change in *CI*s (Change Index) as a function of SOA.** (A)** After PW-paired stimulation, *CI*s in PW or AW were not altered (*p* > 0.05/9 in all conditions, Wilcoxon signed-rank test, Bonferroni correction) across a variety of SOAs. Each data point represents *CI* in the paired direction of every single unit. The error bar represents the mean ± s.e.m. **(B)** After AW-paired stimulation, *CI*s for PW or AW were not altered (*p* > 0.05/9 in all conditions).

We first examined whether SOA influenced the effect of paired stimulation on the response change in the paired direction. The number of single units under each SOA condition was shown in Table 2. The results showed that, in the PW-paired group, *CI*s for PW (Figure 4A, left) or AW (Figure 4A, right) stimulations were not influenced by SOA (*p* = 0.293; *p* = 0.348, Kruskal-Wallis test). Similarly, in the AW-paired group, *CI*s for PW or AW stimulations were not influenced by the SOA as well (*p* = 0.433; *p* = 0.539, Kruskal-Wallis test; Figure 4B). The temporal relationship between the paired optogenetic and mechanical stimuli did not show a predominant influence on the results.

**Table 2:** Number of single units in each pairing parameter combination: 9 SOAs × 2 paired whiskers.

SOA^†^ (ms)	Group	
	PW-paired (*n* = 98)	AW-paired (*n* = 83)
40	16	10
30	2	5
20	20	9
10	12	19
0	8	9
-10	13	11
-20	6	6
-30	10	8
-40	11	6

^†^A negative SOA indicates optogenetic stimulation preceding mechanical stimulation; a positive SOA indicates mechanical stimulation preceding optogenetic stimulation. SOA, stimulus onset asynchrony.

It is interesting to see whether the results using *CI* are different from those using a change of raw spiking rate. The results showed that *CI* and the change of raw spike rate yielded analogous results by showing no significant SOA effects on the change of spiking rate (data not shown) and thus the following analyses were based on *CI*.

### Paired Direction Stimulation Modulates Feature Selectivity When Optogenetic Stimulation Leads Whisker Stimulation

As we found that SOA had no influence on altering neuronal response, it was concerned whether a neuron’s original feature selectivity (direction or orientation tuning) would affect the effect of paired stimulation. To illustrate neuronal direction selectivity to the whisker stimulation, polar plots were constructed to demonstrate the stimulus-driven neuronal responses across directions (Figure 3D). The preferred direction was defined as the direction that had the highest spiking rate. In addition, the direction index (*DI*) and orientation index (*OI*; Pei et al., 2010) were computed to represent the magnitude of feature selectivity of a single unit.

The results showed that the magnitude of the original *DI* did not correlate with *CI* (all *p* > 0.05, Spearman correlation, see Supplementary Figure 1A for details). Similarly, the original *OI* did not correlate with *CI* (all *p* > 0.05, Spearman correlation, see Supplementary Figure 1B for details). These results indicated that the change of *CI* in the paired direction was not affected by the magnitude of original feature selectivity.

**Figure 5:**
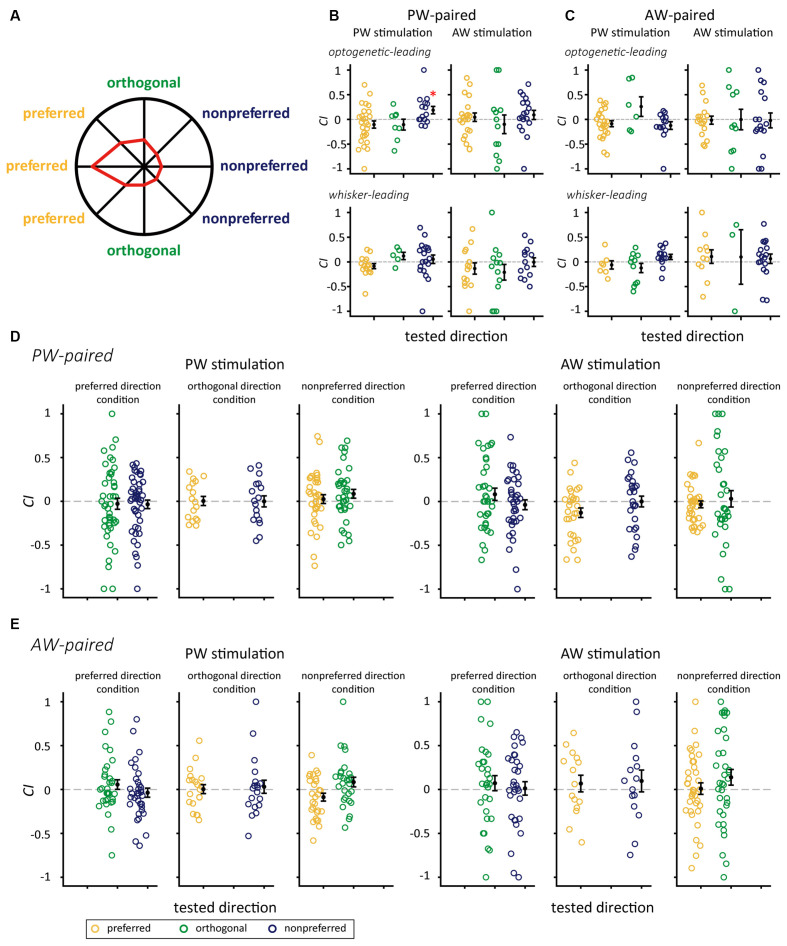
The interaction between paired directions and SOAs on *CI*s for the PW-paired and AW-paired groups. **(A)** The polar plot of directional responses in the sample single unit with the preferred direction of 180°. The preferred, orthogonal, and nonpreferred directions were assigned with respect to its preferred direction. **(B)** The PW-paired group. After paired stimulation, *CI*s for PW increased when paired with the nonpreferred direction in the optogenetic-leading condition, while *CI*s for PW in the mechanical-leading condition and for AW were not altered in any conditions (*for *p* < 0.05/3, Wilcoxon signed-rank test, Bonferroni correction). **(C)** The AW-paired group. *CI*s were not altered in any conditions (*p* > 0.05/3 in all conditions). Yellow, green, and blue circles indicate *CI*s for whisker stimulation in the preferred direction, orthogonal direction, and nonpreferred directions, respectively. **(D)** The PW-paired group. Left panel: *CI*s for PW stimulations in unpaired directions. Right panel: *CI*s for AW stimulations in unpaired directions (*p* > 0.05/2 in all conditions). **(E)** The AW-paired group. Left panel: *CI*s for PW stimulations in unpaired directions. Left panel: *CI*s for AW stimulations in unpaired directions (*p* > 0.05/2 in all conditions).

Additionally, to examine whether a neuron’s preferred direction affect the effect of paired stimulation, we assigned all the conditions in the paired stimulation groups into three relative direction conditions (Figure 5 and Table 3). In the PW-paired group, the optogenetic-leading conditions showed increases in spiking rates that were reflected by positive *CI*s in the nonpreferred direction condition (*CI* = 0.188 ± 0.075, *n* = 15, *p* = 0.011; Wilcoxon signed-rank test, Bonferroni correction) with a median effect size (*r* = 0.408), but not the preferred direction condition (*CI* = −0.104 ± 0.073, *n* = 27, *p* = 0.091) or orthogonal direction condition (*CI* = −0.106 ± 0.110, *n* = 8, *p* = 0.461). In the whisker-leading conditions, *CI*s for PW stimulation did not differ from zero in the three relative direction conditions, including the nonpreferred direction (*CI* = 0.054 ± 0.085, *n* = 19, *p* = 0.158), preferred direction condition (*CI* = −0.086 ± 0.051, *n* = 16, *p* = 0.052), and orthogonal direction conditions (*CI* = 0.120 ± 0.074, *n* = 5, *p* = 0.188).

**Table 3:** Number of single units in each condition: 2 modality sequences × 3 paired directions.

	PW-paired group (*n* = 98)					
Condition^†^	PW stimulation			AW stimulation		
	Preferred (*n* = 46)	Orthogonal (*n* = 17)	Nonpreferred (*n* = 35)	Preferred (*n* = 37)	Orthogonal (*n* = 27)	Nonpreferred (*n* = 34)
Optogenetic-leading	27	8	15	20	12	18
Whisker-leading	16	5	19	14	13	13
	AW-paired group (*n* = 83)					
Condition^†^	PW stimulation			AW stimulation		
	Preferred	Orthogonal	Nonpreferred	Preferred	Orthogonal	Nonpreferred
	(*n* = 35)	(*n* = 20)	(*n* = 28)	(*n* = 32)	(*n* = 15)	(*n* = 36)
Optogenetic-leading	23	6	14	17	10	16
Whisker-leading	7	11	13	11	3	17

*^†^The optogenetic-leading condition includes all negative SOAs; the whisker-leading condition includes all positive SOAs; zero SOA is excluded from both conditions*.

It is important to notice that *CI* of PW response in the PW-paired group differed among paired direction conditions under the optogenetic-leading condition (*p* = 0.043, Kruskal-Wallis test, Figure 5B, top left). Additionally, the *CI* is significantly greater in the nonpreferred direction than that in the preferred direction (*p* = 0.042, *post hoc* Tukey’s HSD test, Figure 5B, top left). In contrast, no effects of paired direction were observed under the mechanical-leading condition (*p* = 0.094, Kruskal-Wallis test; Figure 5B, bottom left). We then examined whether the *CI* difference was simply caused by their intrinsic firing properties or the stimulation of other conditions. The results showed that, before paired stimulation, neurons in the three paired direction conditions showed comparable the spontaneous spiking rate (*p* = 0.417, Kruskal-Wallis test), PW stimulation evoked spiking rate (*p* = 0.605, Kruskal-Wallis test), onset latency (*p* = 0.668, Kruskal-Wallis test), and peak latency (*p* = 0.333, Kruskal-Wallis test; see Supplementary Figure 2 for details).

Similarly, *CI*s for AW stimulation did not differ from zero in any of the factorial combinations of: (1) the optogenetic-leading and whisker-leading conditions; and (2) and relative direction conditions (Figure 5B). Finally, all conditions in the AW-paired group showed *CI*s close to zero (Figure 5C). In summary, the effect of paired stimulation was only observed in the optogenetic-leading, nonpreferred direction condition of the PW-paired group, indicating that neuroplasticity induced by paired optogenetic-mechanical stimulation was limited to specific parameters.

We verified whether the paired stimulation also influenced representations of unmanipulated features and, thus, estimated the *CI*s for the unpaired directions. The results showed that the *CI*s were not significantly different from zero in these unpaired directions, indicating that the effect of paired stimulation did not extend to other stimulus features nor cause a drastic reversal of feature selectivity (Figures 5D,E).

### The Effect of Repetitive Stimulation on Neuronal Responses in the Control Groups

**Figure 6:**
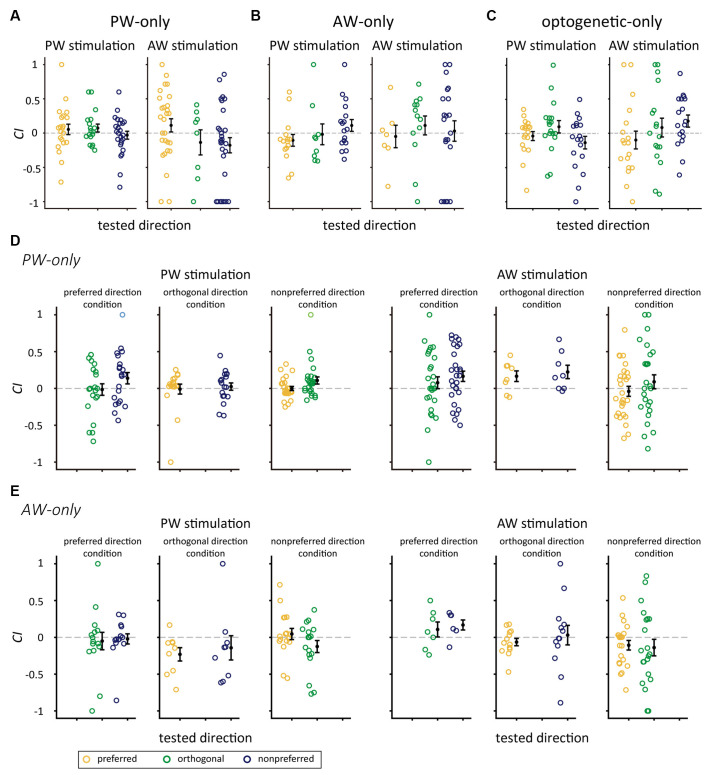
*CI*s for the mechanical-only and optogenetic-only control groups. **(A)***CI*s for the stimulated direction in the PW-only group. **(B)***CI*s for the stimulated direction in the AW-only group. **(C)** In the optogenetic-only group, the *CI*s for PW (left panel) and AW (right panel) stimulation in the preferred, orthogonal, or nonpreferred directions were not altered by repetitive optogenetic stimulation (*p* > 0.05/3 in all conditions, Wilcoxon signed-rank test, Bonferroni correction). **(D)** The PW-only group. *CI*s for whisker stimulation in directions showed in Figure 5A except the repetitively stimulated direction (blank in each panel). Left panel: *CI*s for PW stimulation. Right panel: *CI*s for AW stimulation (*p* > 0.05/2 in all conditions). **(E)** The AW-only group. *CI*s for whisker stimulation in directions showed in Figure 5A except the repetitively stimulated direction (blank in each panel). Left panel: *CI*s for PW stimulation. Right panel: *CI*s for AW stimulation (*p* > 0.05/2 in all conditions). The color convention is the same as in Figure 5.

We examined whether the significant increase in *CI*s could also be observed in repetitive stimulation without pairing. We analyzed the data in the mechanical-only and optogenetic-only groups. The results showed that *CI*s were not altered in any of the relative direction conditions in the PW-only or AW-only group (*p*-value from 0.033–0.947, Wilcoxon signed-rank test, Bonferroni correction; Figures 6A,B, Table 4), or the optogenetic-only group (*n* = 18, *p*-value from 0.071 to 0.879 in all conditions; Figure 6C). Similarly, in the PW-only, AW-only, and optogenetic-only groups, *CI* did not differ across directions (all *p* > 0.05, Kruskal-Wallis test, Figures 6A–C). Furthermore, we examined whether, in the PW-only and AW-only groups, repetitive whisker stimulations in one direction altered neuronal responses in the other directions. The results showed that *CI*s for the directions that were not manipulated by paired or repetitive stimulation did not differ from zero, indicating that neuronal responses were not altered between the prepaired and postpaired periods (Figures 6D,E). *CI* did not differ across directions in the PW-only and AW-only groups (all *p* > 0.05, Wilcoxon rank-sum test, Figures 6D,E).

**Figure 7:**
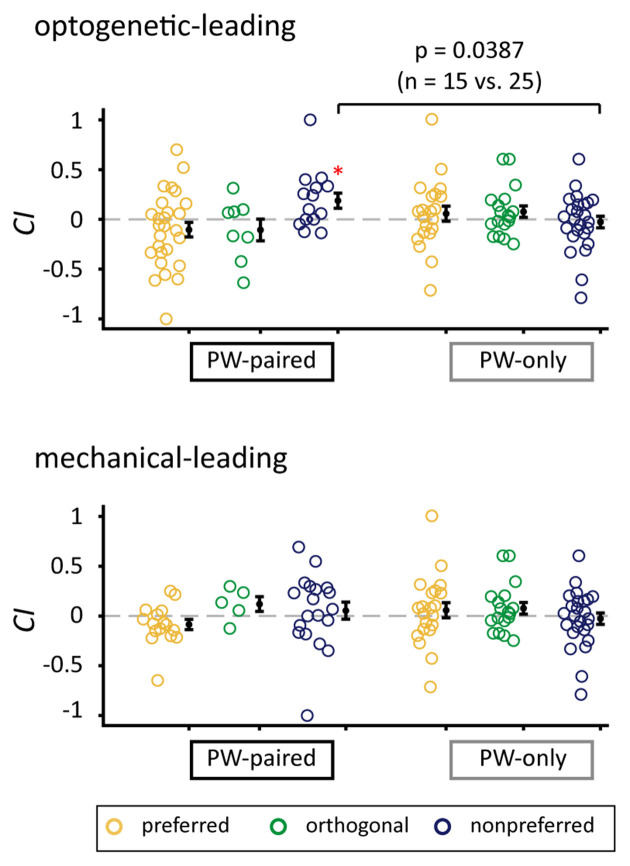
Comparison of *CI*s between the PW-paired and PW-only groups. Comparison of *CI*s for PW stimulation between the PW-paired and PW-only groups. Single units in the PW-paired group receiving the optogenetic-leading (upper panel) and mechanical-leading (lower panel) conditions were analyzed separately. The *CI*s in the nonpreferred condition in the optogenetic-leading condition in the PW-paired group were significantly higher than those in the PW-only group (*p* < 0.05, Wilcoxon rank-sum test). *for *p* < 0.05/3 as in Figure 5B.

*CI*s for the nonpreferred condition in the optogenetic-leading condition in the PW-paired group were significantly higher than those in the PW-only group (*p* = 0.039, Wilcoxon rank-sum test) with a medium effect size (*r* = 0.327; Figure 7), again supporting that the positive *CI*s were induced by the paired stimulation.

**Table 4:** Number of single units in the mechanical-only control groups.

Group	PW stimulation			AW stimulation		
	Preferred	Orthogonal	Nonpreferred	Preferred	Orthogonal	Nonpreferred
PW-only (*n* = 64)	21	18	25	28	8	28
AW-only (*n* = 41)	15	9	17	7	13	21

### Comparison of Spontaneous Neuronal Activities Before and After Paired Stimulation

**Figure 8:**
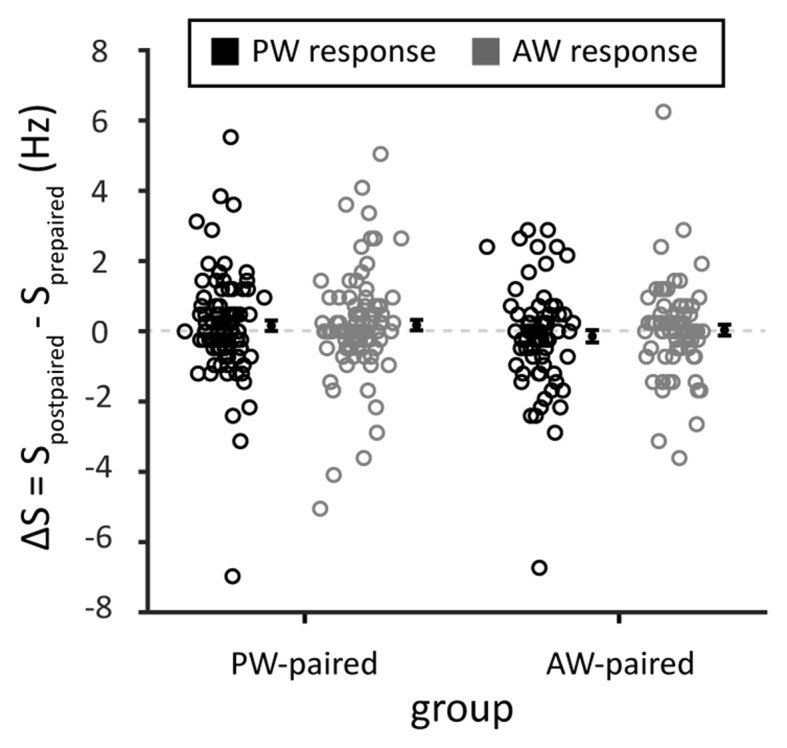
Spontaneous spiking rates before and after the paired stimulation. The mean of the changes in spontaneous spiking rates, obtained from the blank trials among whisker stimulations, before and after paired stimulation (Δ*S* = *S*_postpaired sti._ − *S*_prepaired sti._) was close to zero in any stimulated whiskers or by any whisker that received paired stimulation (*p* > 0.05 in all conditions). *S_postpaired sti._* and *S_prepaired sti._* indicated spontaneous spiking rates before and after the paired stimulation, respectively. Black and gray circles indicate PW and AW stimulations, respectively.

To examine whether paired stimulation alters the spontaneous activities of the recorded single units, we compared the spontaneous spiking rate in blank trials that interleaved between blocks of whisker stimulation. The results showed that the spontaneous spiking rate did not differ before and after paired stimulation (ΔS in PW stimulation, the PW-paired group: 0.16 ± 0.02 Hz, *p* = 0.196, Wilcoxon signed-rank test; in AW stimulation, the PW-paired group: 0.17 ± 0.15 Hz, *p* = 0.172; in PW stimulation, the AW-paired group: −0.14 ± 0.17 Hz, *p* = 0.436; in AW stimulation, the AW-paired group: 0.04 ± 0.15 Hz, *p* = 0.883; comparison between four conditions: *p* = 0.385, Kruskal-Wallis test; Figure 8), indicating that paired stimulation did not alter spontaneous neuronal activities.

**Figure 9:**
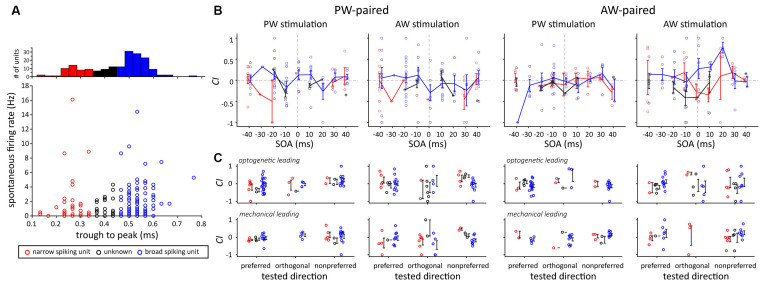
Comparison of *CI*s between two putative neuron types according to their spike shape. **(A)** Neuron type categorization based on their trough-to-peak spike width. Three types were assigned, including the narrow spiking (width <0.35 ms, red dots), broad spiking (width >0.45 ms, blue dots), and unknown (width ≥ 0.35 ms and ≤0.45 ms, black dots) types. **(B)** The effect of SOA did not differ between the two putative neuron types. The error bar represents the mean ± s.e.m. **(C)** The effect of paired direction did not differ between the two putative neuron types.

Finally, we examined whether narrow and broad spiking units had different results in terms of the effect of paired stimulation, as neurons with different spike width and spontaneous spiking rates were shown to have different processing mechanisms (Guo et al., 2014). The results showed that there were no significant differences in terms of their spontaneous activities, the effects of SOA, or the effects of paired stimulation (Figure 9).

## Discussion

The present study indicates that feature selectivity could be altered by activity-dependent modulation using a specific paired stimulation protocol. Indeed, repetitive visual stimulation paired with precise timing of neuronal spikes has been shown to enhance or suppress neuronal responses according to the relative timing between sensory stimulation and cortical spikes (Meliza and Dan, 2006). Analogous approaches that follow the STDP rule were also demonstrated in the auditory (Cruikshank and Weinberger, 2001; D’Amour and Froemke, 2015) and somatosensory systems (Jacob et al., 2007; Litvak et al., 2007). However, other *in vivo* studies revealed a different rule by showing that tuning function was altered only when optogenetic stimulation preceded mechanical whisker stimulation (Khateb et al., 2017; Pauzin et al., 2019). It is noteworthy that although feature selectivity in single neurons could be modulated (Yao and Dan, 2001), the effect of paired stimulation on neuronal populations was reported insignificant in a recent study (Lube et al., 2019). Thus, it is of vital importance to accumulate additional evidence of stimulation-induced neuroplasticity before its clinical application can be fruitfully implemented in the future.

In the present study, we first hypothesized that mechanical stimulation elicits the presynaptic inputs while optogenetic stimulation activates the postsynaptic neuron such that a temporal constraint that follows Hebb’s rule was predicted. However, although optogenetic-mechanical paired stimulation altered neuronal responses exclusively in the paired, nonpreferred direction, its temporal constraints covered a wide range of SOAs, which was incompatible with STDP-based neuroplasticity, in which the facilitation of synaptic strength should peak when postsynaptic spikes occurred approximately 20 ms after EPSP onset (Bi and Poo, 1998; Feldman, 2000; Fino et al., 2008). Interestingly, our results mirror the preliminary findings obtained from the Mormyrid weakly electric fish, showing that neuroplasticity induced by paired stimulation *in vitro* but not *in vivo* is STDP-dependent (Lube et al., 2019).

The optogenetic leading constraint observed in the present study could be accounted for by the supralinear summation of neuronal activities elicited by both whisker and optogenetic stimulation. Khateb et al. (2017) showed that optogenetic stimulation in the motor cortex 0–50 ms ahead of the whisker stimulation could induce supralinear responses to whisker deflections with a sharpening direction selectivity in barrel cortex neurons. Indeed, the nonlinear dendritic processing that receives simultaneous corticocortical and thalamocortical inputs is thought to formulate direction selectivity in the barrel cortex (Lavzin et al., 2012). The wide duration of temporal constraints observed in the present study might thus reflect a long period in which supralinear enhancement could remain after an optogenetic stimulation.

*In vitro* studies have demonstrated that the effect of the synaptic modification is influenced by the initial synaptic connection strength (Bi and Poo, 1998; Sjostrom et al., 2001), in which the synapses with weak strength are more prone to be strengthened. In an *in vivo* study using paired visual stimulation and microstimulation, Meliza and Dan (2006) observed a negative correlation between the magnitude of neuroplasticity and initial synaptic weight. Our results were compatible with this rule: the facilitation of stimulus-driven neural response only occurred in a neuron’s nonpreferred direction when paired stimulation was applied in such a direction.

The present study found that induced alteration of the neuronal response is specific to the manipulated whisker and its paired direction, a finding that is analogous to neuroplasticity induced by sensory adaptation (Katz et al., 2006) and paired stimulation of whisker deflection and current injection (Jacob et al., 2007) in S1BF, in which only neuronal responses associated to a target whisker that received interventions were modulated. In the present study, most stimulation parameters, including a variety of SOAs and paired directions, failed to alter the magnitude of stimulus-driven responses, suggesting that neuronal assemblies in an intact brain are modulated by a variety of balanced inputs (Cauller et al., 1998; Gabernet et al., 2005; Zagha et al., 2013), thus limiting its effect under manipulations.

The usage of optogenetic technique allows us to study stimulation-induced neuroplasticity in specific neural population, namely, putative excitatory neurons carrying the CaMKII promotor in the cortical barrel field (Scheyltjens et al., 2015; Watakabe et al., 2015). Although the present results showed that neuron-type specific manipulation could induce plasticity of neuronal tuning properties, the exact pathway remained unclarified and requires further study. Notably, the transfection volume of the viral vector would not affect the present results because of the cortical area illuminated by the optic fiber was limited (Pisanello et al., 2017). Therefore, we supposed that the effect of paired stimulation was mainly located in the target brain region. However, non-transfected neurons, such as interneurons, may also be sequentially excited by the light-driven neurons. In this sense, our results can only refer to putative neuronal types and further experiments, using juxta-cellular recording or different promotors, are needed to yield neuron-type specific results.

### Limitations

The present study only characterized suprathreshold activities of S1BF neurons; therefore, we could not address the mechanisms relating to subthreshold activities, neurotransmitters, or receptors. Additionally, a neuronal assembly in S1BF might be optogenetically activated as light stimulation was delivered through a nonsheathed optical fiber placed next to the probe (Tamura et al., 2012). Moreover, the connection between putative excitatory neurons in S1BF inside or outside the same cortical barrel (Petersen, 2007) might disrupt or mingle with the bottom-up sensory signal because they were synchronized by ubiquitous illumination. The spatial precision of optogenetic stimulation might not be sufficient to induce the delicate temporal effect known in STDP. Finally, several other factors must be addressed for paired stimulation of neuronal tuning function, such as the dendritic locations relative to the soma (Froemke et al., 2005, 2010), brain regions (Han et al., 2000; Fino et al., 2005; Safo and Regehr, 2008), and cell types (Lu et al., 2007).

### Implications and Future Directions

The present study seeks to develop an animal model to shape functional neuroplasticity *in vivo* using paired optogenetic-mechanical stimulation, an intervention that might benefit patients with neurological disorders. Given that neuroplasticity is shown to account for functional recovery in patients with neurological disorders, such as stroke (Dimyan and Cohen, 2011), the method developed in this study to induce neuroplasticity could thus facilitate the development of clinically feasible interventions in the future. Future works are needed to develop clinically feasible approaches that can be implemented in clinical scenarios.

## Data Availability Statement

The original contributions presented in the study are included in the article/Supplementary Material, further inquiries can be directed to the corresponding author.

## Ethics Statement

The animal study was reviewed and approved by Institutional Animal Care and Use Committee of Linkou Chang Gung Memorial Hospital.

## Author Contributions

Y-PC, J-JH, C-IY, and Y-CP conceived and designed the experiments, interpreted the results of experiments, edited, revised, and approved the final version of the manuscript. Y-PC, J-JH, and Y-CP implemented the setup, performed the experiments, analyzed the data and drafted the manuscript. All authors contributed to the article and approved the submitted version.

## Conflict of Interest

The authors declare that the research was conducted in the absence of any commercial or financial relationships that could be construed as a potential conflict of interest.

## Publisher’s Note

All claims expressed in this article are solely those of the authors and do not necessarily represent those of their affiliated organizations, or those of the publisher, the editors and the reviewers. Any product that may be evaluated in this article, or claim that may be made by its manufacturer, is not guaranteed or endorsed by the publisher.

## Supplementary Material

SUPPLEMENTARY FIGURE 1 Spearman correlation between *CI* and angular selectivity indices before paired stimulation. **(A)** *DI* versus *CI* in the PW-paired (left panels) and AW-paired (right panels) groups. *DI* vs. *CI* in the PW-paired group (*n* = 98), PW stimulation: *r* = −0.095, *p* = 0.354, AW stimulation: *r* = −0.009, *p* = 0.927; in the AW-paired group (*n* = 83), PW stimulation: *r* = 0.123, *p* = 0.267, AW stimulation: *r* = 0.212, *p* = 0.055, Spearman correlation. **(B)** *OI* versus *CI* in the PW-paired (left panels) and AW-paired (right panels) groups. *OI* vs. *CI* in the PW-paired group, PW stimulation: *r* = −0.041, *p* = 0.691, AW stimulation: *r* = −0.026, *p* = 0.798; in the AW-paired group, PW stimulation: *r* = −0.041, *p* = 0.713, AW stimulation: *r* = 0.056, *p* = 0.618, Spearman correlation.

SUPPLEMENTARY FIGURE 2 Spontaneous activity and PW stimulation evoked response properties in optogenetic-leading condition of the PW-paired group before paired stimulation. Spontaneous activity and evoked response, onset latency, and peak latency of PW stimulation from units in three paired direction conditions (*n* = 27, 8, 15, respectively).
