# Supplementary figures and images for: Alternation of Neuronal Feature Selectivity Induced by Paired Optogenetic-Mechanical Stimulation in the Barrel Cortex
Source: Front Neural Circuits. 2021 Sep 1;15:708459. doi: 10.3389/fncir.2021.708459 (PMC8457523; doi:10.3389/fncir.2021.708459)

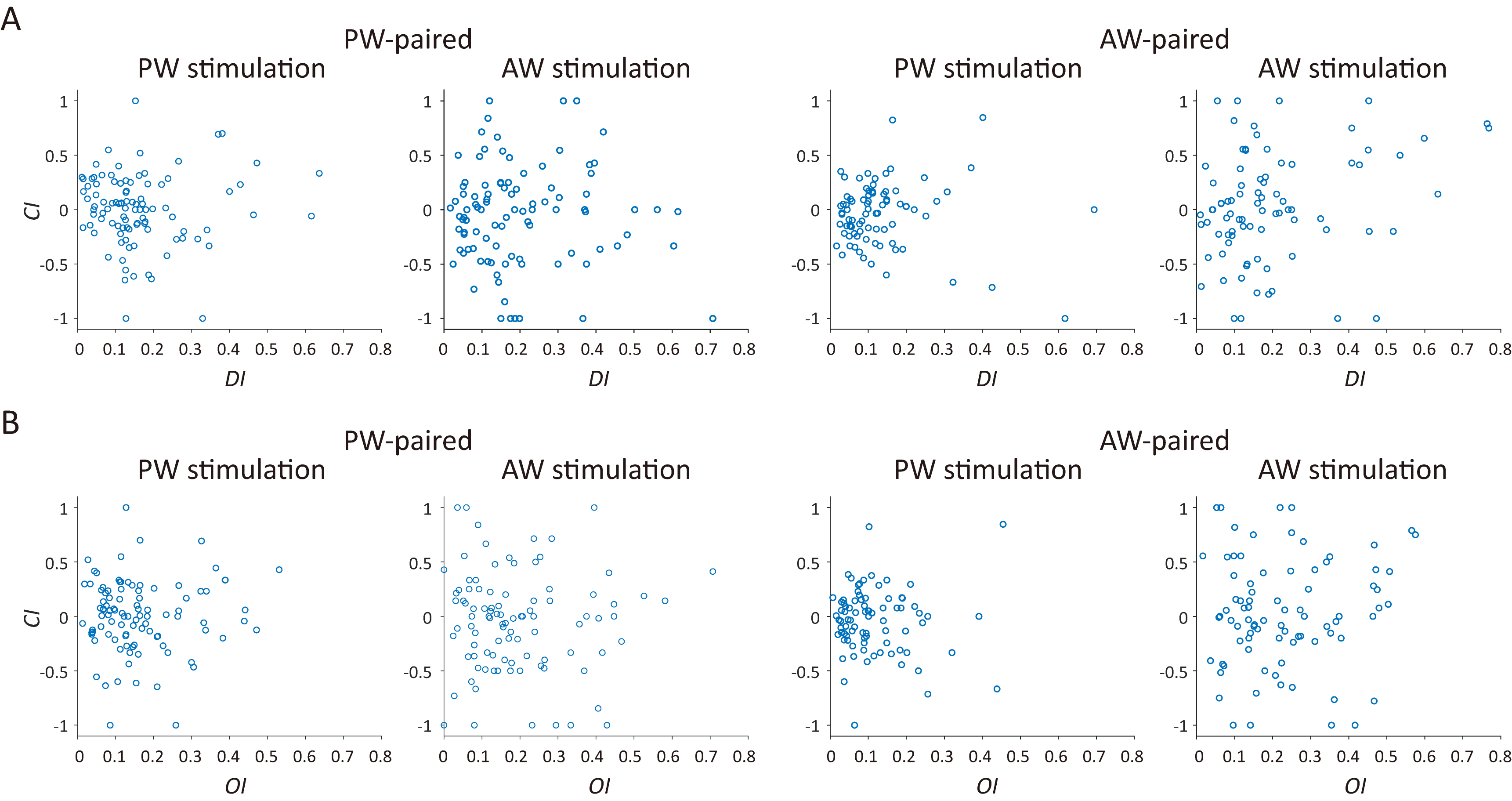

Supplement: SUPPLEMENTARY FIGURE 1 — Spearman correlation between CI and angular selectivity indices before paired stimulation. (A) DI versus CI in the PW-paired (left panels) and AW-paired (right panels) groups. DI vs. CI in the PW-paired group (n = 98), PW stimulation: r = −0.095, p = 0.354, AW stimulation: r = −0.009, p = 0.927; in the AW-paired group (n = 83), PW stimulation: r = 0.123, p = 0.267, AW stimulation: r = 0.212, p = 0.055, Spearman correlation. (B) OI versus CI in the PW-paired (left panels) and AW-paired (right panels) groups. OI vs. CI in the PW-paired group, PW stimulation: r = −0.041, p = 0.691, AW stimulation: r = −0.026, p = 0.798; in the AW-paired group, PW stimulation: r = −0.041, p = 0.713, AW stimulation: r = 0.056, p = 0.618, Spearman correlation. [file Image_1.TIFF]

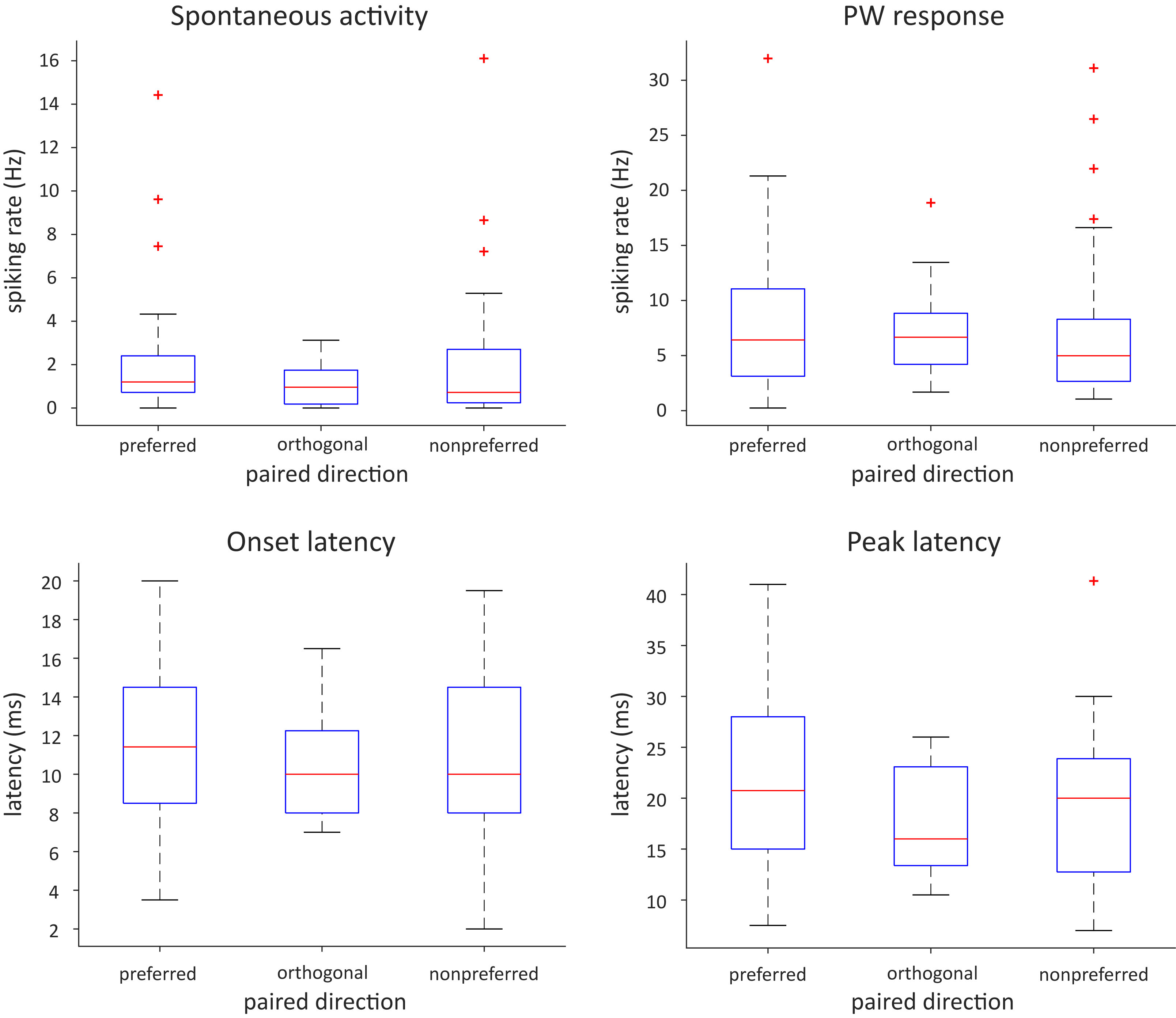

Supplement: SUPPLEMENTARY FIGURE 2 — Spontaneous activity and PW stimulation evoked response properties in optogenetic-leading condition of the PW-paired group before paired stimulation. Spontaneous activity and evoked response, onset latency, and peak latency of PW stimulation from units in three paired direction conditions (n = 27, 8, 15, respectively). [file Image_2.TIFF]
